# Hidden under a Cauliflower-Like Skin Tumor: Chromoblastomycosis

**DOI:** 10.1155/2013/450153

**Published:** 2013-06-20

**Authors:** B. Jakopp, B. Stamm, D. Eyer, A. Conen

**Affiliations:** ^1^Division of Infectious Diseases and Hospital Epidemiology, University Hospital Basel, Petersgraben 4, 4031 Basel, Switzerland; ^2^Division of Infectious Diseases and Hospital Epidemiology, Kantonsspital Aarau, Tellstraße, 5000 Aarau, Switzerland; ^3^Department of Pathology, Kantonsspital Aarau, Tellstraße, 5000 Aarau, Switzerland; ^4^Department of Plastic, Reconstructive and Aesthetic Surgery, Kantonsspital Aarau, Tellstraße, 5000 Aarau, Switzerland

## Abstract

We present the case of an 80-year-old patient with a recurrent hyperpigmented and cauliflower-like skin tumor on the stump of his left index finger. Despite suggestive clinical appearance for chromoblastomycosis the tumor was initially clinically and also histopathologically misdiagnosed as recurrent squamous cell carcinoma. Due to a cardiogenic shock, the patient died shortly after the diagnosis of chromoblastomycosis, before adequate treatment could be introduced. In non-tropical regions chromoblastomycosis is an uncommon chronic fungal infection with *Fonsecaea pedrosoi* being the most prevalent etiological agent. Mostly lower extremities are involved. It is not unusual that, clinically, in the absence of pigmentation, and, histopathologically, because of pseudoepitheliomatous hyperplasia of the epidermis, chromoblastomycosis is confounded with squamous cell cancer, and delays in diagnosis of one to 3 years are common. Therefore, a high grade of clinical suspicion and inclusion of chromoblastomycosis in the differential diagnosis of pigmented skin tumors are important to initiate adequate therapy. Our case is remarkable in many aspects. The localization on an upper extremity and the grade of invasiveness with involvement of bone are unusual; furthermore the lack of a tropical travel history emphasizes that the infection almost surely occurred in Switzerland.

## 1. Introduction

Chromoblastomycosis is a tropical chronic fungal infection of mainly the cutaneous and subcutaneous tissues. It is caused by a pigmented, so-called, dematiaceous fungus, with *Fonsecaea pedrosoi* being the etiological agent in 90% to 96% of cases [1]. Chromoblastomycosis rarely occurs in nontropical regions, and therefore only a few case reports from Europe have been published [2–6]. Most of them are related to tropical travel histories. The fungus originates from the soil and is often associated with thorny plants [1, 7]. Following inoculation by skin trauma, local infection with slowly growing, raised scaly plaques or warty, cauliflower-like lesions develop [7]. There is a predominance of male patients. In 54% to 85% of cases chromoblastomycosis involves the lower extremities, which is explained by the fact that people in tropical regions often walk without shoes or sandals during their fieldwork exposing themselves to the fungus. Although clinical and histological findings are pathognomonic for chromoblastomycosis, confounding with squamous cell carcinoma does occur [1, 7]. Therefore, including chromoblastomycosis in the differential diagnosis of pigmented skin lesions is important to introduce adequate therapy.

We present an unusual case of chromoblastomycosis in a man living in Switzerland without a tropical travel history.

## 2. Case Presentation

**Figure 1:**
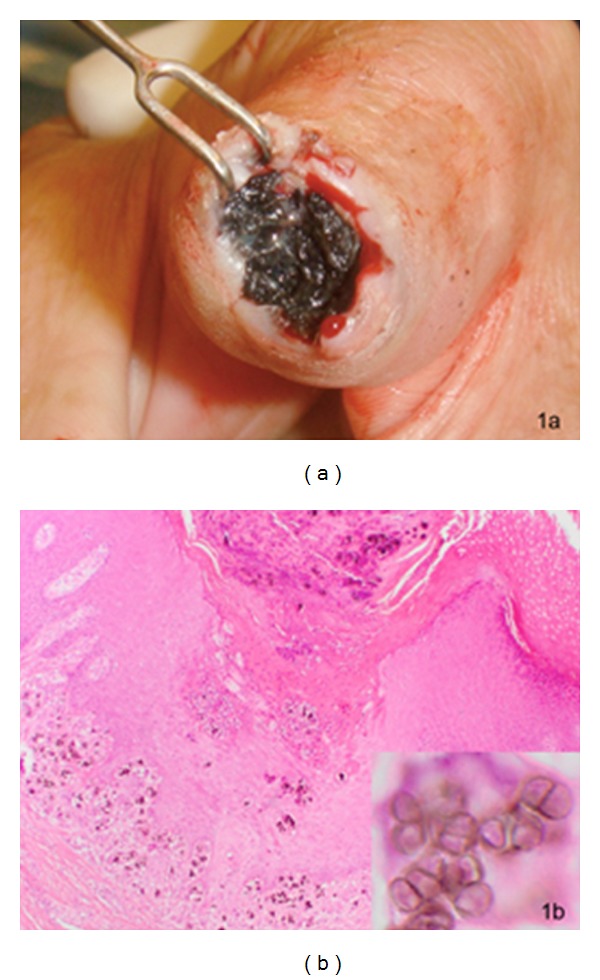
(a) Black pigmented cauliflower-like skin tumor on the amputation stump of the left index finger. (b) Pseudoepitheliomatous hyperplasia and chronic inflammation of the cutaneous, subcutaneous, and osseous tissues (overview) caused by the pigmented fungal elements of chromoblastomycosis (magnification) (hematoxylin and eosin stain).

In May 2010, an 80-year-old man was seen in the infectious diseases outpatient clinic, because of a recurrent black skin tumor on the overwarmed amputation stump of the left index finger. One month before, in April 2010, a black skin tumor on the same location had been surgically removed (intraoperative finding, Figure 1(a)). The patient had no systemic symptoms such as weight loss or fever and was not taking any immunosuppressive drugs. He remembered no traumatic skin lesion except for minor scratches from gardening many years ago.

In his personal history traumatic amputation of the left distal index finger had occurred thirty years ago while working in a garage. In 2008, a tumor had been removed from this amputation stump, and histological analysis revealed a squamous cell carcinoma, which at this time was determined to be cured by the surgical excision. Furthermore, his personal history contained an aortic valve stenosis, chronic renal failure grade 3, and suspected pulmonary metastases of an unknown primary tumor, which was not treated. Originating from the northern part of Italy, he had lived in Switzerland for more than 40 years, and, apart from growing up in Italy, he had not travelled.

In April 2010, using hematoxylin and eosin stain, the histology of the excised tissue had revealed a tumor with marked pseudoepitheliomatous hyperplasia and the presence of numerous chest-nut pigmented, round to oval, thick-walled, occasionally septated fungal elements, so-called sclerotic bodies, forming small clusters and being diagnostic for chromoblastomycosis (Figure 1(b)). The fungus had invaded skin, subcutaneous tissue, and trabecular bone, inducing a chronic, partly neutrophilic, and partly granulomatous inflammation, with many giant cells containing the fungus. Retrospectively the histology of the first tumor excision in 2008 was revised and diagnosed as chromoblastomycosis.

At the follow-up visit in May 2010 surgical revision with radical bone debridement and microbiological diagnostics including fungal cultures was planned. Unfortunately the patient died shortly before due to a cardiogenic shock in June 2010. 

## 3. Discussion

Chromoblastomycosis is an endemic fungal infection in tropical and subtropical regions where most published cases come from [1, 7, 8]. Cases from Europe are rare and often related to a tropical travel history or to work with tropical woods [2–6]. Our patient had neither of both and infection almost surely occurred in Switzerland, which is very unusual. We would like to highlight published cases with the above mentioned exposition sources, such as a Portuguese man who presented with a tumor on the right thigh. He had professional contact with exotic woods [2]. A travel history was found for an English woman who presented with a large plaque on her right forearm 4 months after holiday in Malta [3] and furthermore two cases from France, including a man who immigrated from the Isle de Mayotte and presented with a lesion on his left knee and a woman who had lived in French Polynesia before and presented with a lesion on the right calf [4, 5]. In 9 Finish patients diagnosed with chromoblastomycosis between 1953 and 1963, an association with the hot atmosphere in Finish saunas was discussed, which might have enabled the fungus to grow [6].

Not only the negative travel history to tropical regions is remarkable in our case but also the involvement of a finger and not the lower extremities as typically described [1, 7]. Furthermore we found invasion of the bone which is rarely mentioned in the literature, most recently by de Guzman and Hubbard [9]. We postulate that immunosuppression associated with age, chronic renal failure, and the suspected metastatic tumor disease as well as the missing compacta in the partially amputated phalanx of the index may have enabled the high grade of invasiveness and the atypical localization.

Histopathology is pathognomonic for chromoblastomycosis, but the identification of the causative fungal species can only be obtained by culture. Because of the unexpected death due to a cardiac event, we were not able to get the additional microbiological diagnosis. Delays in diagnosis of chromoblastomycosis of one to 3 years are not unusual, as in our patient (two years). Chromoblastomycosis can be confounded with squamous cell cancer, especially if there is clinical absence of pigmentation and if in histopathology there is a pseudoepitheliomatous hyperplasia of the epidermis [1, 7]. Therefore, a higher grade of clinical suspicion and a wider differential diagnosis of the pigmented skin tumor would have been needed to initiate adequate therapy. On the other side, long standing infection can result in malignant transformation into squamous cell carcinoma [10].

Eradication of chromoblastomycosis is difficult, especially in case of bone invasion [9, 11]. A combination of local physical (cryo- and thermotherapy, liquid nitrogen), surgical, and systemic antifungal treatment with either itraconazole (200–400 mg daily) or terbinafine (500–1000 mg daily) for prolonged periods of time (6–12 months) is most effective. 

In summary, we present a Swiss patient with chromoblastomycosis on the stump of his index finger. Despite the suggestive clinical manifestation with a cauliflower-like growth and hyperpigmentation, there was initially clinical and histopathological misinterpretation as recurrent squamous cell carcinoma, which led to a delay in diagnosis of chromoblastomycosis by 2 years. The localization on an upper extremity, the grade of invasiveness with involvement of bone, and the lack of a tropical travel history are remarkable, especially as infection almost surely occurred in Switzerland. Possible explanations could be that climate changes allow the emergence of this tropical fungus in nontropical regions and that the use of tropical woods in our regions might spread the fungus to our lines of latitude.
